# A Personalized Glomerulus Chip Engineered from Stem Cell-Derived Epithelium and Vascular Endothelium

**DOI:** 10.3390/mi12080967

**Published:** 2021-08-16

**Authors:** Yasmin Roye, Rohan Bhattacharya, Xingrui Mou, Yuhao Zhou, Morgan A. Burt, Samira Musah

**Affiliations:** 1 Department of Biomedical Engineering, Pratt School of Engineering, Duke University, Durham, NC 27708, USA; yasmin.roye@duke.edu (Y.R.); rohan.bhattacharya@duke.edu (R.B.); xingrui.mou@duke.edu (X.M.); yuhao.zhou487@duke.edu (Y.Z.); morgan.burt@duke.edu (M.A.B.); 2 Center for Biomolecular and Tissue Engineering, Duke University, Durham, NC 27708, USA; 3 Division of Nephrology, Department of Medicine, Duke University School of Medicine, Durham, NC 27708, USA; 4 Department of Cell Biology, Duke University, Durham, NC 27708, USA

**Keywords:** human induced pluripotent stem cells, podocytes, endothelial cells, kidney glomerulus, disease models, organ-on-a-chip, glomerulus chip, stem cell technologies, microfluidics, personalized medicine

## Abstract

Progress in understanding kidney disease mechanisms and the development of targeted therapeutics have been limited by the lack of functional in vitro models that can closely recapitulate human physiological responses. Organ Chip (or organ-on-a-chip) microfluidic devices provide unique opportunities to overcome some of these challenges given their ability to model the structure and function of tissues and organs in vitro. Previously established organ chip models typically consist of heterogenous cell populations sourced from multiple donors, limiting their applications in patient-specific disease modeling and personalized medicine. In this study, we engineered a personalized glomerulus chip system reconstituted from human induced pluripotent stem (iPS) cell-derived vascular endothelial cells (ECs) and podocytes from a single patient. Our stem cell-derived kidney glomerulus chip successfully mimics the structure and some essential functions of the glomerular filtration barrier. We further modeled glomerular injury in our tissue chips by administering a clinically relevant dose of the chemotherapy drug Adriamycin. The drug disrupts the structural integrity of the endothelium and the podocyte tissue layers, leading to significant albuminuria as observed in patients with glomerulopathies. We anticipate that the personalized glomerulus chip model established in this report could help advance future studies of kidney disease mechanisms and the discovery of personalized therapies. Given the remarkable ability of human iPS cells to differentiate into almost any cell type, this work also provides a blueprint for the establishment of more personalized organ chip and ‘body-on-a-chip’ models in the future.

## 1. Introduction

More than 15% of U.S. adults have chronic kidney disease (CKD), and many people are unaware that they have the disease due to the lack of early diagnostic tools or methods [1]. CKD progresses into end-stage kidney disease, which is estimated to increase in the US by 11–18% by 2030, partly due to an increased prevalence of diabetes and hypertension—the leading causes of CKD [2,3,4]. For patients suffering from CKD, the quality of life (including physical and mental health) is greatly decreased [5]. Despite technological improvements, dialysis and transplantation are still the only treatment options for kidney dysfunction. Dialysis requires an average of four-hour treatment sessions three times a week, and kidney transplantation has a waitlist with a median wait time of 3.6 years to receive the first kidney [2]. Scientists and clinicians hope to provide additional treatment options, including the ability to regenerate or repair damaged kidney tissues. To achieve these goals and reduce the burden of CKD, researchers are dedicating efforts to the discovery of signaling pathways and molecular targets to help develop novel therapies. The development of robust research platforms and functional in vitro models could greatly benefit from the ability to recapitulate patient-specific biological responses as needed for personalized medicine and related applications [6]. There is also a need to develop in vitro models that are ethical and employ noninvasive technologies to more closely reflect a patient’s response to treatment at the kidney’s cell, tissue, and organ levels. This advancement could help improve patient outcomes by providing a robust preclinical platform for patient-specific disease modeling and mechanistic studies, nephrotoxicity testing, drug screening, and efficacy testing toward the discovery of novel and targeted therapeutics.

As the kidney’s glomerular filtration rate is the most common measure of kidney function, the glomerulus is a critical target for therapeutic development (National Kidney Foundation). The glomerulus is the workhorse of the kidney, filtering about 200 L of blood per day. Each glomerulus consists of a cluster of capillaries lined by fenestrated endothelium. A specialized epithelial cell type named podocyte encases these capillaries. Podocytes have foot processes which interdigitate to form a molecular sieve, called the slit diaphragm. Blood gets filtered through the glomerular filtration barrier, which is composed of fenestrated endothelium, glomerular basement membrane (GBM), and the slit diaphragm of the podocytes. Many kidney diseases, including podocytopathies and glomerulopathies, are characterized by podocyte foot process effacement, actin cytoskeletal remodeling, or detachment of podocytes from the GBM [7]. Such cellular and tissue defects contribute significantly to the progression of kidney disease, to proteinuria, and eventual organ failure.

Although animal models are widely used to study human kidney biology and for preclinical drug development, such models often yield results that are not directly applicable to humans due to species-specific differences in biochemical, physiological, developmental, and anatomical characteristics. Furthermore, normal adult or embryonic human tissues are usually unavailable in sufficient amounts for research, and many mouse models [8,9,10] do not reliably reproduce human phenotypes or biological responses. As a result, it was found that 89.5 % of preclinical drugs that passed animal testing failed during in-human trials [11]. Additionally, heterogeneity in disease phenotypes and varied response to drugs in different patients emphasize the need for more personalized disease modeling approaches. Miniaturized multicellular static culture models (i.e., kidney organoids, transwells, and conventional 2D systems) are useful for understanding nephrogenesis, but these stochastic models often represent the developmental state of first- or second-trimester kidneys, thus lacking the structure and functional characteristics of the specialized kidneys (e.g., vascularization), as well as mechanical factors that promote maturation and in vivo-like phenotypes [12,13,14].

To address these challenges, organ-on-a-chip technologies have been developed to modulate multitissue organization and structure with dynamic control of biochemical cues, vascular perfusion, cellular crosstalk, and mechanical forces to help recapitulate in vivo-like features and functions. For example, cytoskeletal rearrangement and junctional injury in both podocytes and endothelium due to hypertension in a mouse-specific glomerulus chip have been demonstrated [15]. In another study, a proximal tubule chip was employed for high-throughput drug screening while controlling fluid flow and temperature for long-term primary human cell culture [16]. Overall, kidney chips (e.g., glomerulus and proximal tubule) have begun to unveil mechanisms of mechanical- and drug-induced injury in a controlled microenvironment. However, limited supply and invasive sourcing of cells, along with ethical concerns regarding the use of lab animals or human adult primary and embryonic stem cells, limits the potential of the organs-on-chips to mimic in vivo tissue development, physiology, and function. Advancements in human induced pluripotent stem (iPS) cell technologies and the derivation of human mature kidney podocytes have provided novel opportunities to study human kidney diseases without needing to use ethically controversial tissues [17,18,19,20]. Human iPS cell-derived podocytes also allow for studying tissue function and disease processes that are representative of the donor patient’s genetic background.

To date, a human kidney glomerulus chip that consists of genetically matched podocytes and endothelium (thus where both the podocytes and endothelium are derived from the same patient or stem cell line) has not been reported. Such personalized or genetically matched tissue model could advance personalized medicine in the field of nephrology and beyond. By using primary-sourced cells, human kidney glomerulus chips are limited by their difficulty to propagate and/or maintain molecular and genetic characteristics after multiple passages [21,22,23]. To address these issues and further advance organ-on-a-chip technologies and kidney disease outcomes, we present the first glomerulus chip that interfaces epithelial and endothelium tissue layers derived from a single patient’s human iPS cell line. Thus, we engineered a personalized glomerulus chip, which can provide opportunities for patient-specific disease modeling, nephrotoxicity testing, and related personalized medicine applications. By reconstituting multiple glomerular cell and tissue types from a single human iPS cell source, the personalized glomerulus chip provides the aforementioned advantages in uniquely modeling the molecular and genetic profile of a given individual. In addition, this work provides the first demonstration that vascular endothelial cells derived from human iPS cells can be interfaced to model the structure and function of the kidney’s glomerular filtration barrier in vitro.

In this study, we initially differentiated both vascular endothelial cells and intermediate mesoderm cells (nephron progenitor cells) from the same human iPS cell line. We then incorporated these two cell types in interfacing fluidic channels separated by a porous membrane, where the intermediate mesoderm cells were subsequently induced to differentiate or specialize into podocytes within the urinary compartment of the microfluidic chips, while the endothelial cells were propagated in the vascular channel. Together, these human iPS cells generated a personalized patient-specific glomerulus chip. The structural and cellular maturity of the chip (e.g., formation of tissue layers and podocin-positive foot processes) were confirmed through expression of immunofluorescent markers. The functionality of the chip (e.g., secretion of VEGF-165 or VEGF-A and selective molecular filtration across the glomerular filtration barrier) was also investigated. Given our ability to reconstitute the kidney’s glomerular filtration barrier, we also employed the engineered system to model drug-induced injury and disease phenotype. The drug-treated glomerulus chip showed a drastic increase in albumin clearance, indicating loss of barrier function. This device is advantageous in modeling salient features of an individual’s glomerular filtration barrier function, disease phenotype, and response to drugs.

## 2. Materials and Methods

### 2.1. Human iPS Cell Culture

DU-11 (Duke clone #11) human iPS cell line was obtained from Dr. Nenad Bursac’s lab at Duke University under appropriate material transfer agreements and approved by all involved institutional review boards. DU-11 cells were maintained under feeder free conditions in polystyrene tissue-culture-treated plates (Corning, cat. no. 353046) coated with hESC-grade Matrigel (BD Bioscience; 354277). Cells were fed daily with mTeSR1 (Stem Cell Technologies; 85850) medium. These cells were passaged every 4–5 days (upon 70–80% confluency) by treatment with StemPro Accutase (Thermo/Life Technologies; A1110501). DU-11 cells have been tested for and found to be free of mycoplasma contamination (using Mycoplasma PCR Detection Kit from abm, G238). Chromosomal analysis confirmed that these cells were karyotypically normal.

### 2.2. Intermediate Mesoderm Cell Differentiation

DU-11 cells (70–80% confluent) were incubated with enzyme free cell dissociation buffer (Gibco, 13150-016) for 15 min. The dissociated cells were scraped off the well and centrifuged at 201× *g* in Advanced DMEM/F12 (Gibco; 12634010) for 5 min. The cells were seeded on Laminin-511-E8 (Iwai North America, cat. no. N-892012) coated plates with mesoderm induction media as reported earlier. [19,20,24] After 2 days of differentiation, the mesoderm cells were further differentiated into intermediate mesoderm (IM) cells in IM induction media for 14 days.

### 2.3. Vascular Endothelial Cell (viEC) Differentiation

viEC were differentiated from iPSCs via previously established protocol (Atchinson et al., 2020). On day 0, iPSCs were harvested with Accutase and reseeded on Matrigel-coated six-well plate at a cell density of 47,000 cells/cm^2^. On day 1, mTeSR1 media was replaced with N2B27 media, which contains neurobasal media (Invitrogen) and DMEM/F12 glutamax (Invitrogen) in 1:1 ratio with N2 (100×) (Invitrogen) and B27 (-) Vitamin A (Invitrogen). The media was then supplemented with 8 µM CHIR-99021 (Reprocell, Inc., Beltsville, MD, USA), and 25 ng/mL hBMP4 (VWR International LLC, Radnor, PA, USA). Cells were cultured in N2B27 media without media changing for 3 days to achieve lateral mesoderm induction.

On Day 4, N2B27 media was replaced with endothelial induction media which contained StemPro-34 SFM media (Invitrogen) supplemented with Glutamax (Invitrogen) and Pen-Strep (Gibco Cell Culture) in 100:1:1 ratio, and supplemented with 2 µM forskolin (Abcam Inc., Cambridge, UK) and 200 ng/mL VEGF165 (Invitrogen). Media was changed daily from Day 4 to Day 6 and conditioned media was collected on Day 5 to Day 7. viEC were then dissociated on Day 7 with Accutase, and sorted to harvest CD144+ (VE-Cadherin) and CD31+ (PECAM-1) cells, as described below.

### 2.4. viEC Magnet-Activated Cell Sorting

Cells collected on Day 7 of the vascular endothelial differentiation were individualized and completely dissociated (incubated at 37 °C for 5–7 min) with Accutase. Cells were collected and neutralized with cold StemPro-34 media in 1:1 ratio and centrifuged at 1000 rpm for 5 min. After washing with 10 mL of MACS buffer, which consists of DPBS (Duke University Cell Culture Facility, Durham, NC, USA) with 0.5% BSA (Sigma Aldrich, Saint Louis, MO, USA), and 2 mM EDTA (Invitrogen), cells were resuspended in MACS buffer at the density of 80 µL/10 million cells, followed by 20 µL/10 million cells conjugation with FcR blocking reagent, CD31 Microbeads, and CD144 Microbeads (Miltenyi Biotec, Inc., Bergisch Gladbach, Germany). The cell suspension was incubated for 15 min on ice in the dark. After ice-incubation, cells were washed with MACS buffer and sorted on QuadroMACS™ Separator via the magnetic-mediated approach. Sorted cells were expanded in conditioned media diluted at 1:1 ratio with StemPro-34 SFM and supplemented with 2 µg/mL heparin (STEMCELL Technologies, Vancouver, BC, Canada). Media was replaced every other day until conditioned media was depleted. For continued expansion after passage 1, viEC maintenance media was prepared as StemPro-34, supplemented with 10% HI-FBS (Invitrogen), 2 µg/mL heparin, and 50 ng/mL VEGF165.

### 2.5. Glomerulus Chip Device Functionalization and Cell Seeding

Organ-chip devices were purchased from Emulate. Inc. (Boston, MA, USA). The chips were activated/sterilized in a plasma etcher (Emitech K-1050X) by treatment with oxygen plasma (100 W, 0.8 mbar, 30 s). The activated chips were immediately coated with 50 μg/mL of laminin-511 solution (BioLamina, Sundbyberg, Sweden; LN511-0502) in PBS with calcium and magnesium in their urinary and microvascular channels (separated by a porous PDMS membrane) and incubated overnight at 37 °C.

The channels of the chips were then flushed with Advanced DMEM/F12 (Gibco, MA, USA; 12634010). Differentiated viECs (9 × 10^4^) were seeded in the microvascular channel of the chips and incubated by inversion. After 3 h, the channel was flushed with viEC maintenance media to remove the non-adherent cells, and incubated overnight at 37 °C. Next, the differentiated IM cells (8 × 10^4^) were seeded in the urinary channel. After 3 h, both fluidic channels were flushed with their respective media (Urinary channel—IM induction medium and microvascular channel—viEC maintenance medium) and incubated overnight at 37 °C.

### 2.6. Personalized Glomerulus Chip Culture Maintenance

The urinary and the microvascular channel reservoirs of the Emulate Pod were filled with podocyte induction media and viEC maintenance media, respectively. The Pods were primed for 2 min to ensure fluid flow in the inlet and the outlet of the fluid circuit. Both channels of the chips were flushed with their respective media (Urinary channel—IM induction media and microvascular channel—viEC maintenance media) and then connected to the Pods while maintaining fluid–fluid contact to avoid bubble formation. The Pods were placed inside an automated vacuum regulator (Orb) that also directs fluid flow and controls stretch–relaxation cycles. The chip channels were continuously perfused with the respective cell culture medium at a volumetric flow rate of 60 µL/h which correlates with a shear stress of 0.0007 dyn/cm^2^ for the urinary channel and 0.017 dyn/cm^2^ for the microvascular channel, along with cyclic strain (10% at 0.4 Hz) through the two vacuum channels parallel to the fluidic compartments of the chip [19,20]. The stretch and relaxation cycle of the porous membrane mimics the mechanical strain exerted by renal blood flow and pressure in vivo. After 5 days of podocyte differentiation, the podocyte induction media was replaced with CultureBoost-R (Cell Systems SF-4Z0-500-R) and the resulting glomerulus chip was maintained for an additional 7 d.

### 2.7. Functional Analysis of the Glomerular Filtration Barrier

Spent medium (CultureBoost-R) was collected from the top (urinary channel) outlet reservoir of the glomerulus chips after completion of the podocyte differentiation. The level of cell secreted VEGF-A was quantified using the human VEGF-A ELISA kit (Thermo Fisher Scientific; Waltham, MA, USA, BMS277-2) per the manufacture’s protocol. A parametric two-tailed Student’s *t*-test was used to calculate significant differences between two groups. All error bars represent standard deviation of the mean, unless otherwise noted. For all statistical analyses, the GraphPad Prism 9 software package was used.

### 2.8. Immunostaining and Microscopy

The bright field images were captured using an EVOS M7000 microscope. For immunostaining, the cells were fixed, permeabilized, and blocked using our earlier method [19,20]. The primary antibodies used included Nephrin (ARP, MA, USA; GP-N2), Podocin (Abcam, MA, USA; ab50339), VE-cadherin (Santa Cruz, Dallas, TX, USA; SC-9989), PECAM (R&D Systems, Minneapolis, MA, USA; AF-806), Alexa Fluor 594 Phalloidin (Invitrogen, Waltham, MA, USA, A12381), and Collagen IV (eBioscience, Waltham, MA, USA, 14-9871-82). Immunostained chips were imaged with a Leica SP8 Upright Confocal Microscope, using a 25×/0.95 HCXIRAPO water dipping lens with 2.4 mm DIC objective. Images were processed using Fiji software version 2.1.0/1.53c. Analysis of the DU-11 podocyte arborizations at the podocyte-endothelial cell interface in the glomerulus chips was analyzed using the 3D viewer plugin of Fiji software (version 2.1.0/1.53c).

### 2.9. Modeling Drug-Induced Nephrotoxicity

After podocyte induction, the chips were maintained in CultureBoost-R (Cell Systems SF-4Z0-500-R) for at least 5 days under fluid flow and mechanical strain before introduction of Adriamycin (LC Laboratories, Woburn, MA, USA; D-4000). viEC maintenance media was supplemented with clinically relevant doses [19,24] of Adriamycin (0.5 µg/mL) and perfused through the microvascular channel for 48 h. Next, the viEC maintenance media was supplemented with 100 μg/mL albumin conjugated to Alexa Fluor 555 (Thermo Fisher Scientific; A34786) and perfused through the microvascular channel. 

To study the extent of the disruption of the glomerular filtration barrier, urinary clearance of albumin was evaluated in the glomerulus chip. Media was collected from the top outlet reservoir, and fluorescence intensity was measured using a SpectraMax Fluorescent Plate reader (SpectraMax i3x, Molecular Devices). The amount of Albumin filtered from the microvascular to the urinary channel was analyzed using an equation for renal clearance: ([U] × UV)/[P]) where [U] is urinary concentration of albumin, UV = volume of media collected from the urinary channel outlet reservoir, and [P] = dosing concentration in the microvascular channel (100 μg/mL). A parametric two-tailed Student’s *t*-test was used for calculating significant differences between two groups. All error bars represent standard deviation of the mean, unless otherwise noted. For all statistical analyses, the GraphPad Prism 9 software package was used.

## 3. Results

### 3.1. Derivation of Intermediate Mesoderm and Vascular Endothelial Cells from Human iPS Cells

Previously established kidney glomerulus chip devices employed podocytes and endothelial cells derived from different sources and individuals, and thus these organ-on-a-chip devices contained tissue layers with unmatched genetic backgrounds. Some of the sources of cells for these glomerulus chip devices include animal or non-human sources [15,25], primary human podocytes [26], primary human glomerular endothelium [20], amniotic fluid-derived podocytes [26], and human iPS cell-derived podocytes [19]. While the use of animal cells is limited in physiological translation into humans, even the use of primary, immortalized, or amniotic fluid-derived cells are limited in their multipotency and/or availability. We used previously established methods for the derivation of podocytes and endothelial cells [19,20,27,28,29].

**Figure 1:**
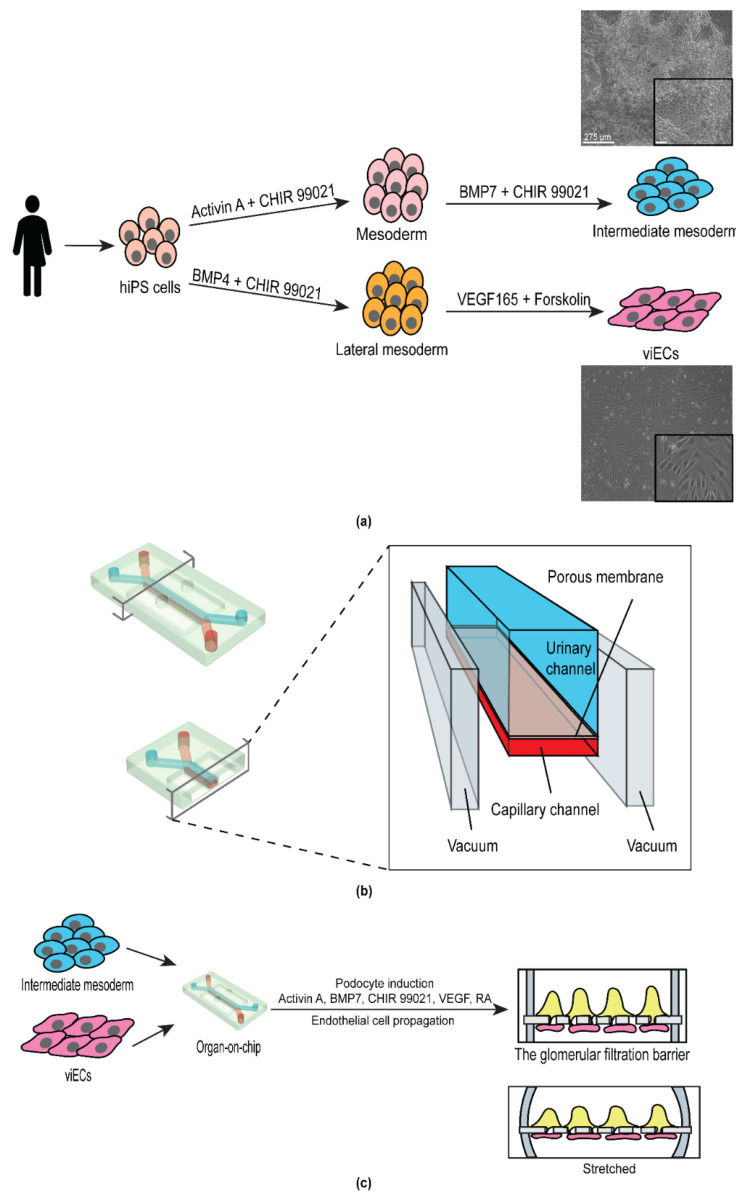
Schematic of the experimental strategy for engineering a personalized glomerulus chip. (**a**) Derivation of intermediate mesoderm and vascular induced endothelial cells from human induced pluripotent stem cells derived from a single individual. Images on the right are representative bright field images of intermediate mesoderm (top) and vascular induced endothelial cells (bottom). Scale bar 275 μm. Inset scale bar: 90 µm; (**b**) Anatomy of the glomerulus chip. Lower left panel, cross-sectional view of the chip. Right panel, channel anatomy of the chip; and (**c**) Incorporation of iPS cell-derived intermediate mesoderm and endothelial cells into the urinary and the microcapillary channel of the chip, respectively and subsequent differentiation of mature human podocytes from intermediate mesoderm cells. Upper right panel, cross-sectional view of the cell-embedded glomerulus chip. Lower right panel, cross-sectional view of the cell-embedded glomerulus chip under stretch.

DU-11 human iPS cells produced from a single donor were used to derive human intermediate mesoderm (IM) and vascular endothelial cells (viECs) (Figure 1a). For the derivation of IM cells, we first induced the formation of mesoderm cells using a medium containing Activin A and the Wnt agonist CHIR99021. We further induced posteriorization of the mesoderm cells using Bone Morphogenetic protein-7 (BMP7) and Wnt agonist CHIR99021. BMP7 signals through the SMAD1/5 axis which helps the formation of IM cells (mesenchymal Nephron progenitor cells), whereas CHIR99021 primes their mesenchymal to epithelial transition (MET) [19,20,26].

The protocol of Atchison et al. (2020) was used to differentiate the same DU-11 human iPS cell line into viECs in 7 days [28] (Figure 1a). Briefly, human iPS cells were differentiated into lateral mesoderm lineage by using N2B27 media supplemented with Wnt agonist CHIR 99021 and BMP4. Vascular endothelial cells were then induced from the lateral mesoderm cells by treatment with STEMPro-34 SFM medium supplemented with VEGF-A (angiogenesis inducer) and Forskolin (cyclic AMP booster). Cells were sorted based on expression of platelet endothelial cell adhesion (PECAM-1, CD31) and/or vascular endothelial cadherin (VE-Cadherin, CD144).

### 3.2. Design and Development of the Personalized Glomerulus Chip

For the development of the personalized glomerulus chip, we utilized polydimethylsiloxane (PDMS) microfluidic chips from Emulate, Inc. (Boston, MA, USA). The chip includes two parallel fluidic channels positioned on top of each other to recapitulate the body’s dynamic tissue–tissue interface. The top (urinary) and the bottom (capillary) channels are separated by a 50 µm thick flexible PDMS membrane. The membrane consists of hexagonally packed pores with approximately 7 µm diameter which are spaced 40 µm apart from each other. We functionalized the PDMS chip with Laminin-511 as the extracellular matrix protein on both channels to allow two interfacing cell types to anchor onto the porous membrane. Upon cell seeding in the respective channels, the level of shear stress generated by the fluid flow was determined by the flow rate (60 µL/h) and chip dimensions. The capillary channel experienced higher shear stress owing to its smaller dimensions (shear stress = 0.017 dyn cm^−2^, 1000 µm × 200 µm (w*h)) compared to the urinary channel (shear stress = 0.0007 dyn cm^−2^, (1000 µm × 1000 µm (w*h)) [27]. On each side of the two fluidic channels were hollow vacuum chambers that enabled cyclic stretching and relaxation cycles, thereby mimicking the pressure generated by pulsatile blood flow in the kidneys (Figure 1b).

From a structural standpoint, the glomerulus is a tuft of capillaries that are encased by terminally differentiated epithelial cells called podocytes, and the lumen is lined by endothelial cells. We seeded our differentiated viECs in the capillary channel to mimic the endothelial circuit of the glomerulus. To form the epithelial layer, we first seeded IM cells in the urinary channel, then differentiated them into podocytes within the glomerulus chip device using podocyte induction medium (Figure 1c). This podocyte induction medium has been shown to generate podocytes with mature and functional phenotypes [19,20]. One limitation of using human iPS cells is the lack of functional maturation in their derivative cells. However, it has been shown that small molecules can enhance the expression of mature endothelial and epithelial phenotype and function [19,20,28,29]. Our tissue model developed specialized phenotypes and expressed markers of mature cell types in presence of the soluble growth factors, cell secreted soluble morphogens, fluid flow, and mechanical stimulation, as previously demonstrated by Ingber and colleagues [19,20].

### 3.3. Differentiated Human iPS Cell-Derived Podocytes and Vascular Endothelium Emulate the Structure of the Kidney Glomerulus

**Figure 2:**
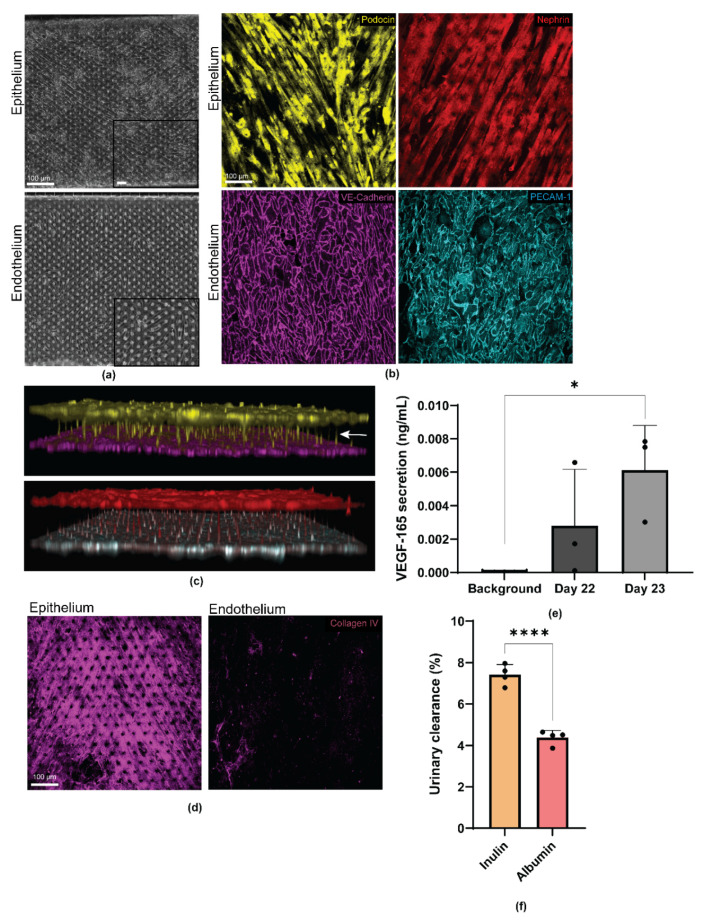
Human iPS cell derived glomerular epithelial (podocytes) and endothelial cells express mature cell markers and form foot processes extending from podocytes to endothelial cells, indicating intercellular cross talk. (**a**) Bright field images of the glomerular epithelia in the urinary channel and endothelium in the capillary channel. Scale bar 100 µm; (**b**) Representative confocal microscopy images of the podocytes and endothelial cells stained for Podocin (yellow), Nephrin (red), VE Cadherin (magenta), and PECAM-1 (cyan), respectively. Scale bar 100 µm; (**c**) Three-dimensional reconstruction of the epithelial to endothelial tissue–tissue interaction formed by Podocin positive (yellow) podocytes and VE Cadherin positive (magenta) endothelial cells; and Nephrin positive (red) podocytes and PECAM-1 positive (cyan) endothelial cells at the interface; arrow indicates cellular crosstalk through physical proximity; (**d**) Representative confocal microscopy images of collagen IV positive (magenta) glomerular epithelia in the urinary channel and endothelium in the capillary channel. Scale bar 100 µm; (**e**) Quantification of the VEGF-165 secretion by the mature podocytes. Background indicates culture medium not exposed to podocytes. *n* = 3. * *p* = 0.0169 and (**f**) Quantification of percent inulin and albumin clearance from the urinary channel of the glomerulus chip. *n* = 4. **** *p* = 0.0001.

Brightfield images of the capillary and urinary channels of the organ chip revealed uniform cell monolayers formed by the iPS cell-derived podocytes and endothelial cells in their respective channels (Figure 2a). The endothelial cell layer in the capillary channel expressed VE-Cadherin and PECAM-1 that were predominantly localized to the cell junctions (Figure 2b, Endothelium). To determine the maturation state of the podocytes, we immunostained the urinary channel with the podocyte-specific markers, nephrin, and podocin. These proteins were used to characterize the podocytes and their foot processes. Confocal microscopy analysis revealed patterned cellular distribution and alignment of the podocytes in the direction of fluid flow. The resulting podocytes also expressed key lineage specific markers associated with the mature phenotype. The podocytes formed a barrier with an arborized network, which is synonymous with the glomerular filtration barrier (Figure 2b, Epithelium).

In an intact and functional kidney, podocytes develop foot processes that form interdigitations with adjacent podocytes to form the glomerular filtration sieve or network. To examine whether such structure could form in our engineered device, we captured a confocal z-series of our glomerulus chips. As shown by the 3D reconstruction of the confocal microscopy images, human iPS cell-derived epithelium and endothelium formed confluent monolayers on their respective sides of the porous PDMS membrane. Additionally, podocytes in the urinary channel were found to extend their cellular processes through the PDMS membrane pores and towards the direction of the endothelial cells in the capillary channel—a process reminiscent of intercellular crosstalk through physical proximity. Interestingly, we observed that podocin expression was largely localized to the cell bodies of the podocytes, as would be expected for specialized podocytes (Figure 2c). In addition to epithelium and endothelium, the glomerular filtration barrier is also comprised of the glomerular basement membrane (GBM), which separates the two cell types. The GBM contributes to the size selectivity of the glomerular filtration barrier, as well as providing significant structural integrity [30,31,32]. Amongst many proteins in the GBM, type IV collagen is the most abundant (~50%) [33]. Mostly produced by podocytes (and less so by endothelium), type IV collagen plays an essential role in the generation, organization, and maintenance of the GBM in addition to being an indicator of proper cellular crosstalk [34,35]. Confocal microscopy analysis of our engineered glomerulus chips revealed type IV collagen secretion by both epithelium (podocytes) and endothelium (Figure 2d, Supplemental Figure S1A) with expression highest in the urinary channel or by the podocytes. The sandwich-like layering of the three-component system (consisting of epithelium (podocyte), basement membrane, and endothelium) recapitulates the structure of the glomerular capillary wall or blood filtration barrier.

Given the phenotypic maturation of iPS cell-derived podocytes and endothelial cells in the glomerulus chip, we next analyzed the functional characteristics of the podocytes. During glomerulogenesis, VEGF-A is secreted by the metanephric mesenchyme to direct the migration of endothelial cells towards the developing glomerulus [36]. Mature kidney podocytes produce VEGF-A throughout their life span to help maintain homeostasis and the function and phenotype of the capillary endothelial cells [37,38]. We quantified VEGF-A secretion from the differentiated podocytes, and found significantly high levels of VEGF-A (VEGF-165 isoform) secretion (*p* = 0.0169), with the most optimal secretion timepoint occurring on day 23 (Figure 2e). Furthermore, we quantified the selective molecular size filtration of the personalized glomerulus chip through inulin and albumin urinary clearance. It was found that the small molecule, inulin, was excreted significantly more than the large protein, albumin, as seen in vivo (*p* = 0.0001) (Figure 2f). Taken together, the successful establishment of our personalized glomerulus chip indicates that single donor iPS cell-derived podocyte and endothelium can serve as an in vitro model of normal glomerular phenotype and function.

### 3.4. Personalized Glomerulus Chip Models Drug-Induced Nephropathy

**Figure 3:**
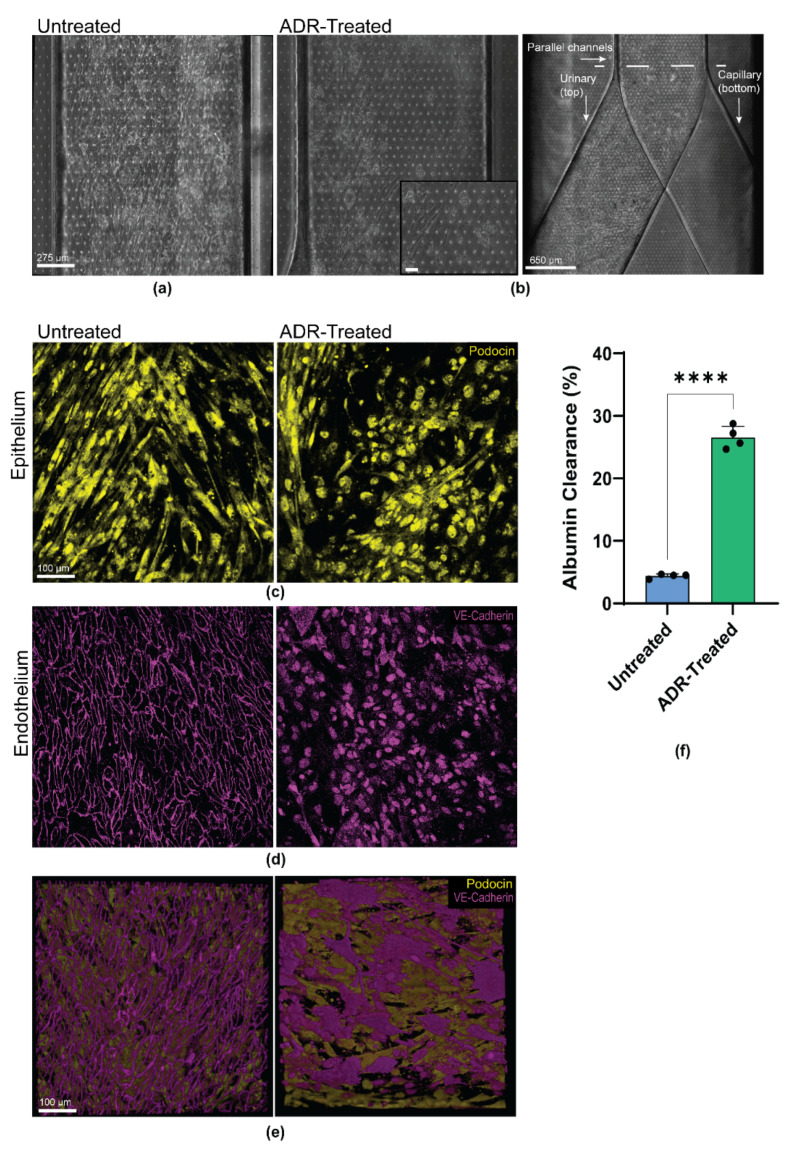
Personalized glomerulus chip recapitulates Adriamycin-induced kidney injury. (**a**) Brightfield images of the glomerular epithelium in the urinary channel of the control/untreated. Scale bar: 275 µm; (**b**) Brightfield images of the glomerular epithelium in the urinary channel of the treated glomerulus chip after 48 h of Adriamycin. Scale bar: 275 µm and 650 µm. Dashed line indicates point where individual channels merge. Inset: Delaminated and effaced podocytes in the urinary channel. Scale bar: 90 µm; (**c**) Representative confocal microscopy images of the untreated (left) and treated (right) glomerulus chip Podocin positive (yellow) epithelium in the urinary channel. Scale bar 100 µm; (**d**) Representative confocal microscopy images of the untreated (left) and treated (right) glomerulus chip VE-Cadherin positive (magenta) endothelium in the capillary channel. Scale bar 100 µm; (**e**) Three-dimensional overlay of Podocin-positive epithelium and VE-Cadherin positive endothelium of the untreated (left) and treated (right) glomerulus chip. Images represent basal to apical view. Scale bar 100 µm; and (**f**) Quantification of percent albumin clearance from the urinary channel of the glomerulus chip 24 h post Adriamycin treatment compared to the untreated chip. *n* = 4. **** *p* = 0.0001.

The chemotherapy drug Adriamycin (ADR) has been used as a model of nephrotoxicity in vivo and in vitro [24,39]. We examined whether the engineered patient-specific glomerulus chip could model tissue toxicity and disease phenotype when treated with ADR. The clinically relevant dose of ADR, 0.5 μg mL^−1^ [19] was continuously perfused through the capillary channel of the chip, mimicking intravenous administration of the drug for up to 48 h. Injury within the glomerulus chip was noted, as the endothelium in the capillary channel were almost completely detached. In contrast, the podocytes that were interfaced with the capillary channel showed more significant signs of injury (Figure 3a,b). These results suggest that the podocytes were able to respond to the nephrotoxic drug, which potentially caused the loss of cell structure integrity and network, resulting in subsequent podocyte delamination and foot process effacement. The podocytes retracted their foot processes from the pores of the PDMS membrane and were unable to re-establish the intact cell structure necessary for selective molecular filtration across the glomerular capillary wall (Figure 3c, Supplemental Figure S2A). Prior to ADR treatment, podocyte cytoskeleton (F-actin) aligned with the direction of fluid flow. As ADR was administered in the capillary channel, podocyte actin cytoskeletal rearrangement was observed in the podocytes interfacing with the endothelial cells exposed to the drug (Supplemental Figure S2B). Podocyte injury was also exacerbated by the increased vascular permeability whereby the cell culture medium from the capillary channel migrated at a higher rate to the urinary compartment due to loss of barrier function caused by ADR treatment. We observed the remodeling of VE-Cadherin and PECAM-1 by loss of intercellular junctional localization in the endothelial cells exposed to ADR (Figure 3e, Supplemental Figure S2C). An overlay of the glomerulus chip barrier showing the respective cell types and their expression of lineage markers reveal damage to both the endothelium and epithelium after ADR perfusion, and the cellular damage was visibly more significant in the endothelium within the capillary channel (Figure 3f, Supplemental Figure S1D). It was also observed that the middle of the capillary channel showed less endothelial detachment than the ends of the channel. Where the endothelium was interfaced with podocytes in the chip, the podocytes were most injured. Damage to the once confluent monolayer of vascular endothelial cells exposes podocytes to the drug, and decreases size selectivity of the glomerular filtration barrier due to epithelial delamination and effacement. To further examine the loss of size-selective filtration barrier, we introduced 100 μg mL^−1^ of fluorescently labelled albumin to the capillary channel of the chip after ADR perfusion. We observed a significant increase in the urinary clearance of albumin compared to the control (*p* = 0.0001), successfully recapitulating drug-induced albuminuria (Figure 3f).

## 4. Discussion

### 4.1. The Personalized Glomerulus Chip

Traditional tissue culture vessels have been useful in studies of basic biological processes, but these culture systems often fail to accurately model tissue-specific functions or predict in vivo relevance of drug candidates. Animal models have significantly contributed to the field of drug development and toxicity analysis, but researchers have realized the developmental and metabolic discordances between animal and human studies that necessitates establishment of patient-specific platforms to more closely predict human physiologicalresponses. In this study, we engineered a personalized human in vitro model of the glomerulus, a microcapillary-like system lined by epithelial and endothelial cells important for establishing the blood filtration barrier and selective molecular filtration function of the kidney. Development of personalized in vitro models of human tissues and organs could improve the 90% failure rate of clinical trials and minimize ethical concerns surrounding the use of animal models. Emerging organs-on-chips technologies are promising for pharmacokinetics/pharmacodynamic modeling [40]. Furthermore, these microphysiological platforms are being used to discover novel therapeutic targets [41]. Incorporation of the human blood filtration system into multi organ-on-chip models is essential for having an accurate account of the drug’s interaction. Deriving the glomerular filtration barrier tissues from a single patient not only preserves the innate crosstalk between cell types, but also harnesses the proliferative power of human iPS cells to serve as an unlimited supply of specialized cell types to model and predict the drug response of a specific individual or patient.

The kidney plays an important role in fluid hemostasis and drug excretion. Drug-induced nephrotoxicity impairs intraglomerular hemodynamics, which affects 20% of the adult population in the United States [1,2]. Methods for assaying drug toxicity based on simple cell culture assays containing immortalized podocytes fail to faithfully predict the effects of drugs in humans. Given the ability to model some aspects of the glomerular filtration barrier, we studied the effects of the chemotherapeutic drug, Adriamycin, in the personalized glomerulus chip. We continuously perfused Adriamycin for up to 48 h in the vascular channel of the glomerulus chip and observed massive endothelial cell death with intercellular junctional remodeling and hypertrophy. As the dying vascular endothelial cells continued to detach from the porous PDMS membrane, podocytes in the top channel became increasingly exposed to the drug, thus undergoing cell injury, characterized by loss of foot processes, retraction of cellular structures, and eventual cell detachment.

### 4.2. Model Limitations and Outlook

Our model provides a low-cost approach that can be used in the future to stratify the subpopulation of patients who respond differently to drugs or present distinct mechanisms of disease. These personalized glomerulus chips can be engineered as “you-on-a-chip” systems, to study different disease states and drug responses, treatment comparisons, and dosage optimizations for patients with debilitating kidney-related chronic conditions such as hereditary podocytopathies, cardio-renal diseases, and immunological effects of organ transplantation. Patients with rare genetic nephropathies could benefit from these injury/disease-on-a-chip methods to help unveil developmental mechanisms and identify novel treatment options. Additionally, acquired diseases, such as cardiorenal- and kidney-brain axis neurological disorders are becoming more relevant with the increase in epidemiologic data on CKD and related secondary disorders [42,43]. The increase in venous pressure severely affects the mechanisms and outcomes of these extra-renal complications, yet there is still limited knowledge on its affects in the human glomerulus’ structure and function. Elucidating the mechanobiology of venous pressure in a controlled microenvironment is difficult to do in animal models such as mice, but is well-suited for the personalized glomerulus chip, as flow rate, channel shear stress, and mechanical forces can be dynamically regulated. Additionally, the transparency of the PDMS-based personalized glomerulus chip makes it more relevant than animal models to study real-time tissue-drug interactions and dynamics of the glomerulus using fluorescence microscopy. In addition, drug toxicity often has an immunological component. Hence, including immune cells in these perfusable organ chips could aid in the assessment of the immune and inflammation responses (i.e., cytokine storm) in response to biologics or viral infections. Furthermore, the PDMS membrane (50 µm in thickness) used in this study is much thicker than the native glomerular filtration barrier (approximately 315 nm) [19] and PDMS is non-biodegradable. Thus, future work focusing on integrating thin biodegradable materials in the microfluidic chip to serve as an alternative to the PDMS membrane could further advance the function and physiological relevance of the patient-specific glomerulus chips. Successful derivation of individualized organ chips with a single human iPS cell patient donor, as demonstrated in this study, could help advance personalized medicine approaches and avoid mixed results by ensuring a consistent genetic background that more accurately represents a patient’s biological responses.

## Data Availability

The data that support the findings of this study are available from the corresponding author upon a reasonable request.

## Supplementary Materials

The following are available online at https://www.mdpi.com/article/10.3390/mi12080967/s1, Figure S1: Human iPS cell derived epithelium and endothelium secrete glomerular basement membrane protein, collagen type IV; Figure S2: Human iPS cell-derived epithelium (podocyte layer) and endothelium recapitulate Adriamycin-induced tissue damage and actin cytoskeletal remodeling.
