# Supplementary material for: A Personalized Glomerulus Chip Engineered from Stem Cell-Derived Epithelium and Vascular Endothelium
Source: Micromachines (Basel). 2021 Aug 16;12(8):967. doi: 10.3390/mi12080967 (PMC8400556; doi:10.3390/mi12080967)
Supplement: Supplementary file 1 [file micromachines-12-00967-s001.zip › micromachines-1291434-supplementary.pdf]

*Supplementary:*

# A Personalized Glomerulus Chip Engineered from Stem Cell-Derived Epithelium and Vascular Endothelium

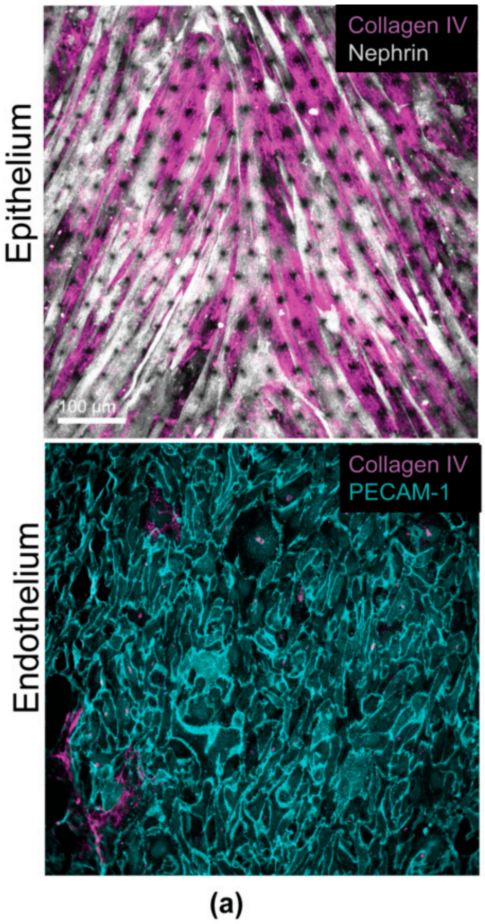


**Figure S1.** Human iPS cell-derived epithelium and endothelium secrete the most abundant glomerular basement membrane protein, collagen type IV. (a) Representative confocal microscopy images of the collagen type IV-positive (magenta), Nephrin-positive (white) epithelium (top), collagen type IV-positive (magenta), and PECAM-1-positive endothelium (bottom). Scale bar 100 µm.

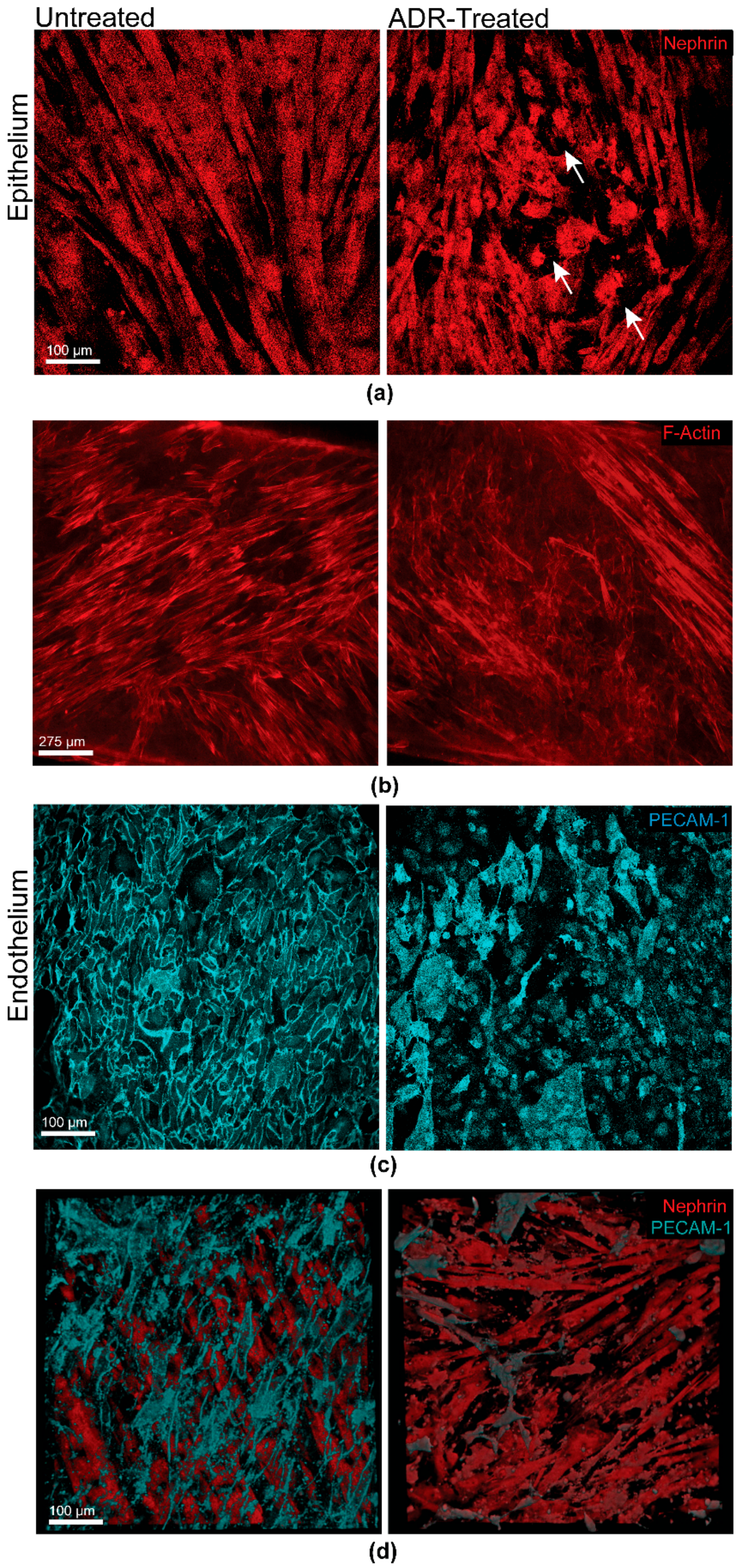


**Figure S2.** Human iPS cell-derived epithelium (podocyte layer) and endothelium recapitulate Adriamycin-induced tissue damage. (**a**) Representative confocal microscopy images of the untreated and treated Nephrin positive (red) podocytes in the urinary channel. Arrows indicate specific podocytes' cell body retraction. Scale bar 100 µm; (**b**) Representative immunofluorescent images of the untreated and treated F-actin positive (red) podocytes in the urinary channel. Scale bar 275 µm (**c**) Representative confocal microscopy images of the untreated and treated PECAM-1 positive (cyan) endothelium in the capillary channel. Scale bar 100 µm (**d**) Three-dimensional overlay of Nephrin positive epithelium and PECAM-1 positive endothelium. Images represent basal to apical view. Scale bar 100 µm.
